# Photosensitizing properties of hollow microcapsules built by multilayer self-assembly of poly(allylamine hydrochloride) modified with rose Bengal†

**DOI:** 10.1039/c9ra03153g

**Published:** 2019-06-18

**Authors:** Mariana P. Serrano, Matías Rafti, Andrés H. Thomas, Claudio D. Borsarelli

**Affiliations:** Instituto de Investigaciones Fisicoquímicas Teóricas y Aplicadas (INIFTA), Departamento de Química, Facultad de Ciencias Exactas, Universidad Nacional de La Plata, CCT La Plata-CONICET La Plata Argentina mariserr@inifta.unlp.edu.ar; Instituto de Bionanotecnología del NOA (INBIONATEC), CONICET. Universidad Nacional de Santiago del Estero (UNSE) RN9, Km 1125 G4206XCP Santiago del Estero Argentina

## Abstract

A polymeric photosensitizer based on poly(allylamine hydrochloride) (PAH) and rose Bengal (RB) was synthesized. The modified polycation PAH-RB was demonstrated to be suitable for construction of microcapsules *via* a layer-by-layer (LbL) assembly technique, using sodium poly(styrene sulfonate) (PSS) as counter-polyelectrolyte and CaCO_3_ microcrystals as templates. After CaCO_3_ core removal, a stable suspension of hollow microcapsules with shells incorporating RB (HM-RB) was obtained. The spectroscopic and photophysical behavior of both PAH-RB and HM-RB in aqueous environments were studied and described in terms of dye–dye interactions and dye hydrophobicity. Only HM-RB was able to generate singlet molecular oxygen with similar efficiency to free RB in air-saturated solutions upon green light irradiation. In order to explore possible practical applications as a supramolecular photosensitizer, experiments of HM-RB irradiation in the presence of chemically and biologically relevant target molecules were carried out. It was observed that is possible to use visible light to initiate the photooxidation of biological compounds in water, with many interesting advantages compared to low-molecular-weight photosensitizers such as an enhancement of the photosensitizing effect, due to a significant reduction of dye–dye interaction, or improved reuse given the straightforward size-based separation from the reaction mixture without loss of efficiency.

## Abstract 2

We explored a novel strategy directed to the improvement of rose Bengal fluorescence emission and photosensitization properties by supramolecular assembly into hollow microcapsules using LbL technique.

## Graphical abstract

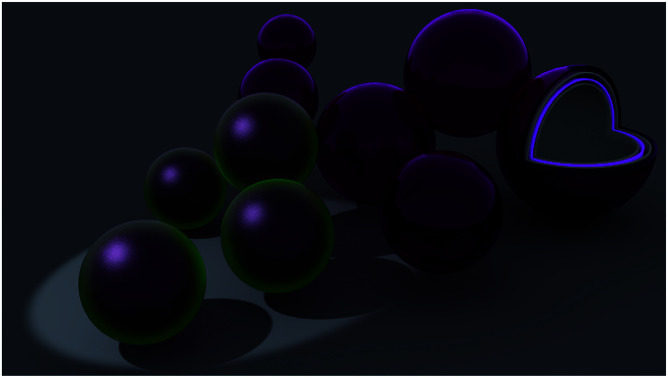

## Introduction

Photosensitized reactions constitute one of the possible practical uses of solar radiation. In these processes, a chemical species called a photosensitizer, absorbs solar radiation in a certain spectral range and induces chemical changes in a target molecule, which does not absorb radiation. Photosensitized processes have a wide range of applications such as synthesis with controlled regio- and stereo-selectivity,^1^ photodynamic therapy (PDT),^2^ photoinactivation of microorganisms (PDI),^3^ and wastewater treatment for pollutant removal.^4^ In general, the photosensitization process may involve two types of mechanism: (i) the generation of radicals (type I mechanism, *e.g.*, *via* electron transfer or hydrogen abstraction); and/or (ii) the production of singlet oxygen (^1^O_2_) through an energy transfer process (type II mechanism).^5–7^

Among the main drawbacks of using homogeneous photosensitized reactions is the non-straightforward removal and reutilization procedures needed for continuous operation. As a consequence, an increasing interest in the development of strategies directed towards immobilization of photosensitizers on different platforms has been recently observed;^8,9^*e.g.*, modification of synthetic and natural polymers with organic dyes were used in the development of new photoactive materials for water treatment,^8,10^ PDI^11^ and PDT.^12^

**Fig. 1:**
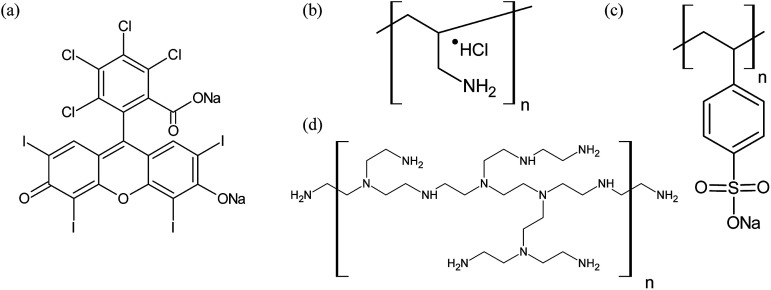
Molecular structure of (a) rose Bengal (RB) and the polymers: (b) poly(allylamine hydrochloride) (PAH), (c) sodium poly(styrene sulfonate) (PSS), and (d) poly(ethyleneimine) (PEI).

Rose Bengal (RB) (Fig. 1) is a xanthene dye intensively used as photosensitizer for the ^1^O_2_ production,^13^ due to its high solubility in water, high radiation absorption in the visible region, and high quantum yield of triplet-state production.^14^ Additionally, RB can be easily anchored to different polymers using; *e.g.*, the method introduced by Merrifield,^15^ which has been already employed in the development of polymer-based photosensitizers for photooxidation.^16–18^ Photocatalytic materials based on immobilized dyes on different platforms were already reported for environmental applications.^19,20^ However, the main drawback in the development of such photosensitizer materials is the tendency to form molecular aggregates driven by dye-to-dye interactions caused by high local concentrations, thus provoking an enhancement of non-radiative deactivation pathways and the consequent decrease of photosensitizing efficiency.^21,22^

It is therefore highly desirable, to attain control over the spatial distribution of dye molecules in order to improve the design of photo-active polymeric materials. Such spatial control can be achieved for films using layer-by-layer (LbL) assembly technique, which offers a simple and versatile construction method based on the sequential adsorption of interacting species; *e.g.*, oppositely charged polyelectrolytes.^23^ Sodium poly(styrene sulfonate) (PSS), poly(ethyleneimine) (PEI), and poly(allylamine hydrochloride) (PAH) are typical synthetic polyelectrolytes used for multilayer formation (Fig. 1).^24,25^ Such films can be conveniently functionalized *via* post-synthetic modification with selected molecules^26^ or nanoparticles,^27,28^ or by using already functionalized polyelectrolytes.^29–31^ LbL assembly can be carried out to create films, but also grown over different templates, such as carbonates microparticles, which after removal with suitable chelating agents, give raise to hollow microcapsules (HM).^32^

Although the use of RB in the assembly of HM was already reported,^30,33^ the influence of different anchoring strategies and its assembly into LbL architectures on their photophysical and photosensitization properties was not studied in detail. The goals of the present work can be summarized as follows: (i) to synthesize a RB modified PAH polycation (PAH-RB); (ii) to use such PAH-RB polycation for microcapsule formation *via* LbL with CaCO_3_ microspheres as sacrificial templates and PSS as counter polyelectrolyte; (iii) to evaluate the effect of polymer conformation and spatial distribution in the photophysical properties of RB; and finally (iv) to assess the photosensitizing properties of such supramolecular assembly using chemical and biological oxidizable targets.

## Experimental methods

### Materials

All reagents were used as received without further purification. Rose Bengal (RB), sodium poly(styrene sulfonate) (PSS, average *M*_w_ ∼ 70 000), poly(allylamine hydrochloride) (PAH, average *M*_w_ ∼ 58 000), 1-ethyl-3-(3-dimethylamino-propyl)carbodiimide (EDC), anthracene-9,10-dipropionic acid disodium salt (ADPA) and 2′-deoxyguanosine 5′-monophosphate (dGMP) were purchased from Sigma-Aldrich (Sigma-Aldrich, Argentina). Poly(ethyleneimine) solution 50 wt% in H_2_O (PEI, average *M*_w_ ∼ 750 000) and *N*-hydroxysulfo succinimide sodium salt (sulfo-NHS) were purchased from Fluka (Argentina). Pro-analysis anhydrous sodium carbonate, calcium chloride dihydrate, sodium chloride, hydrochloric acid, sodium hydroxide, and ethanol were obtained from Cicarelli (Argentina). Ultrapure deionized water was used for all experiments.

### Synthesis of PAH-RB and microcapsule preparation

The EDC/NHS mediated synthesis of PAH-RB was inspired in the bioconjugation and labelling of proteins.^34^ Briefly, EDC and sulfo-NHS solution was added to an RB buffered solution and stirred evenly. Then, 20 mL of PAH solution was added and stirred for 15 h. The final product was purified by dialysis. The whole reaction and dialysis process were protected from light.

**Scheme 1:**
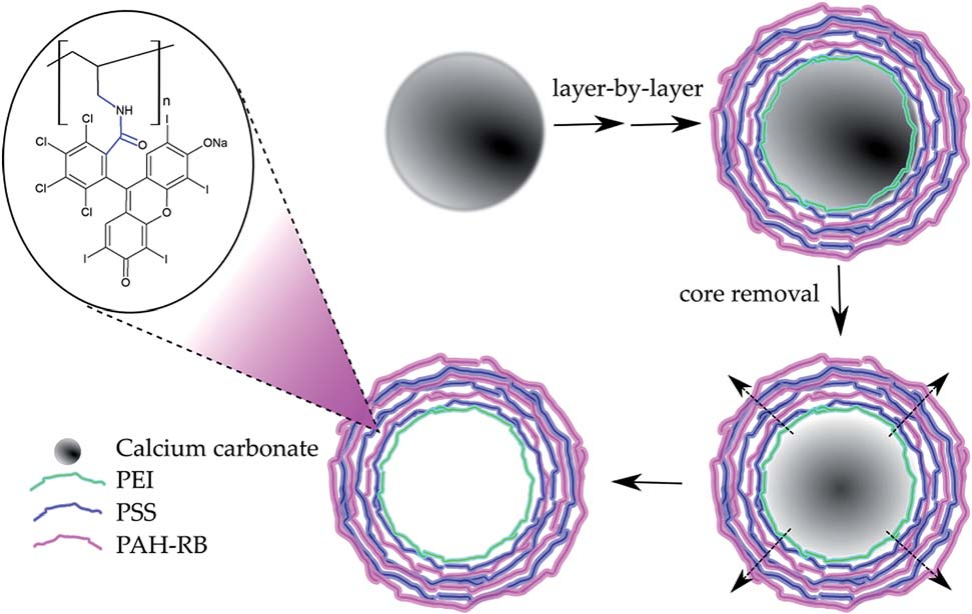
Preparation of hollow microcapsules (HM) using layer by layer self-assembly technique onto CaCO_3_ template. Inset: proposed molecular structure of the PAH-RB polycation.

Polymeric microcapsules were built using CaCO_3_ templates sequentially coated with oppositely charged polyelectrolytes layers (LbL assembly),^23,24^ see Scheme 1 and ESI.† Removal of CaCO_3_ template was carried by re-suspension of the coated microparticles with 0.1 M EDTA solution at pH 7 during 30 min at 25 °C while shaking at 1100 rpm. A final wash was performed using 50 mM NaCl solution to remove the remaining EDTA (Scheme 1). Finally, the obtained HM were stored at 8 °C before use.

### Polymer and microcapsule characterization

#### UV-vis spectroscopy

UV-vis absorption spectra were registered with a Shimadzu UV-1800 spectrophotometer, using quartz cells of 1 cm optical path length. All the absorption spectra registered for HM were corrected using cubic polynomial extrapolations of the spectral baseline in the visible region, between 400–800 nm, as a simple approximation of scattering contribution of the sample.^35^

#### Fluorescence emission

Steady-state and time-resolved fluorescence measurements were performed using a single-photon-counting equipment FL3TCSPC-SP (Horiba Jobin Yvon), described elsewhere.^36^ All the experiments were carried using 1 cm optical path quartz cells. The fluorescence quantum yields (*Φ*_F_) were determined from the corrected fluorescence spectra using eqn (1).1
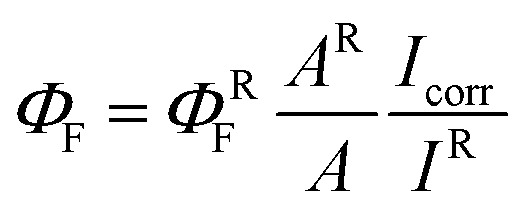
where *I*_corr_ is the corrected maximum intensity, *A* is the absorbance at the excitation wavelength (*λ*_exc_) and the superscript R refers to the reference fluorophore. To avoid inner filter effects, an RB aqueous solution of absorbance below 0.1 at the excitation wavelength was used as fluorescence reference (*Φ*_F_ = 0.018). The *I*_corr_ values were calculated at the maximum of the fluorescence band registered between 530 to 700 nm. The fluorescence intensity from RB in the HM was corrected by scattering using eqn (2).

2 *I*_corr_ = *I*_obs_ × 10^(*A*_exc_+*A*_emi_)/2^

where *I*_corr_ and *I*_obs_ were the corrected and observed fluorescence maximum intensities, *A*_exc_ and *A*_emi_ were the absorbance of the system at excitation and emission wavelengths, respectively.

Fluorescence decays were registered with a time-correlated single photon counting (TSCPC) apparatus from Horiba. In time-resolved experiments a NanoLED source (maximum at 575 nm) was used for excitation and the emitted photons were registered by a TBX-04 detector and counted by a FluoroHub-B module. The fluorescence intensity decays were fitted with the Fluorescence Decay Analysis Software DAS6® from Horiba by deconvolution of the NanoLED pulse using multi-exponential model function.

#### Infrared spectroscopy

Fourier-transform infrared spectra were obtained with a Jasco 4600 spectrometer with an attenuated total reflectance (ATR-FTIR) accessory containing a single reflection monolithic diamond crystal. Spectral resolution was 4 cm^−1^ and background corrections were performed by assuming an average refractive index of 1.45 for all the samples.

#### Dynamic light scattering and zeta-potential

All dynamic light scattering (DLS) and zeta-potential measurements were performed with a cell drive voltage of 3.3 V in 10 mM HEPES NaCl 500 mM buffer solutions at pH 7 and 25 °C, using a Zetasizer Nano ZS apparatus from Malvern.

#### Transient absorption experiments

Laser-flash photolysis (LFP) experiments were performed using a homemade LFP system with laser pulse excitation at 532 nm (7 ns full width at half maximum, 7 mJ per pulse) as previously described.^37^ The transient absorption decays of RB and PAH-RB solutions or HM suspensions were acquired with a Tektronix TDS3032B 300 MHz instrument, and up to 10 single-shot signals averaged. Prior each measurement, the solutions were degassed by Ar bubbling for 20 min. The signal analysis of the transient decays was done with the OriginPro 8.5 software from OriginLab Corporation using a mono and/or bi-exponential fitting function.

#### Fluorescence microscopy

Optical microscopy images were acquired on a Carl Zeiss Axio-Observer Z1 inverted microscope. Samples were illuminated using bright field or an HBO Xe lamp complete with excitation and emission bandpass filters of 550 ± 50 nm and 605 ± 70 nm, respectively, acquired on an AxioCam camera. Zen imaging software was used for image acquisition and analysis. All fluorescence microscopy images were digitally colored post acquisition.

### Steady-state photolysis experiments

Aqueous suspensions containing the target molecule and HM were irradiated in 1 cm optical path quartz cells at room temperature in continuous stirring. Two different irradiation systems were employed: (I) a Xenon arc lamp (300 W, Newport) coupled to a motorized monochromator UV-VIS (Oriel Cornerstone 130 1/8 m) by a Mounting Kit (Model 74017) and (II) a Xenon arc lamp (150 W, Luzchem) with a 475 nm filter (GG475). UV-vis absorption spectral changes of anthracene-9,10-dipropionic acid disodium salt (ADPA) were registered with a CCD-USB2000 UV-vis spectrometer (OceanOptics), with the analyzing beam placed at right angle of the photolysis beam from the irradiation setup (II).

A high-performance liquid chromatograph Prominence from Shimadzu (solvent delivery module LC-20AT, on-line degasser DGU-20A5, communications bus module CBM-20, auto sampler SIL-20A HT, column oven CTO-10AS VP and photodiode array detector SPD-M20A) was employed for monitoring the photo-chemical process of 2′-deoxyguanosine 5′-monophosphate. A Synergi Polar-RP column (ether-linked phenyl phase with polar end-capping, 150 × 4.6 mm, 4 μm, Phenomenex) was used for products separation. Solutions containing 3% of methanol and 97% of 25 mM formic acid (pH = 3.2) were used as mobile phase.

## Results and discussion

### Characterization of PAH-RB polycation

**Fig. 2:**
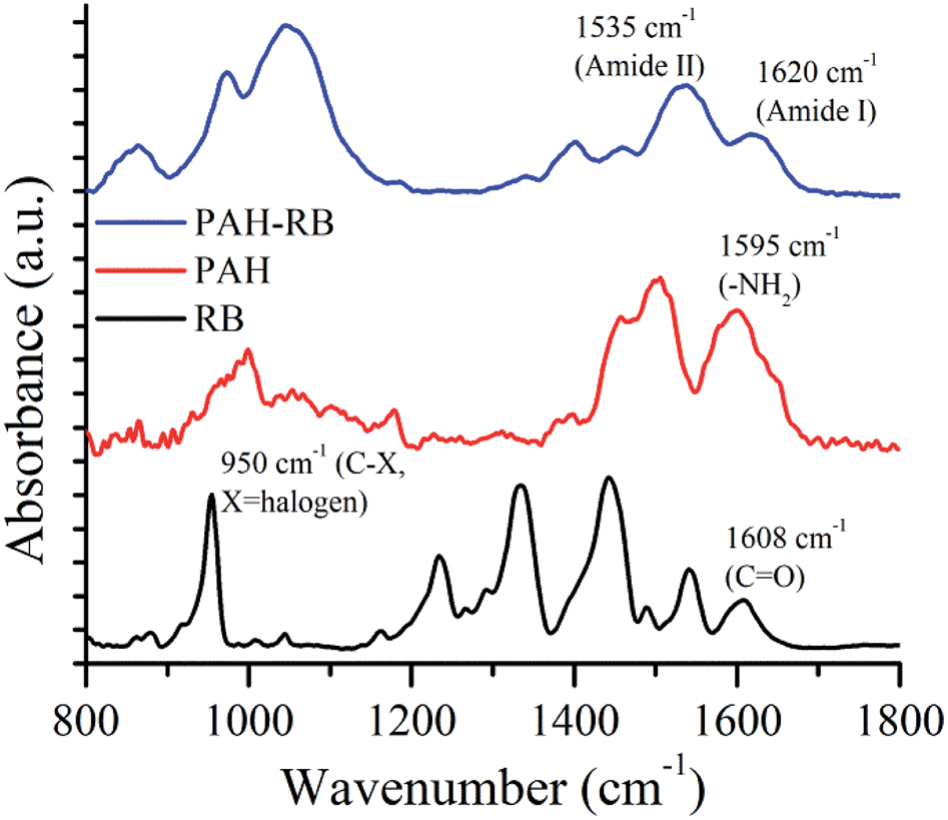
ATR-FTIR spectra for RB, PAH and PAH-RB polycation.

After covalent functionalization of PAH with RB, the resulting PAH-RB polycation was characterized thoroughly, Fig. 2 shows a comparison of ATR-FTIR absorbance spectra of RB, PAH, and PAH-RB. The carbonyl stretching mode corresponding to RB appears at 1608 cm^−1^, whereas absorption peaks at 1543, 1442, and 1334 cm^−1^ correspond to aromatic C=C stretching modes.^38^ For PAH, a signal at 1595 cm^−1^ corresponding to N–H in-plane bending mode appears. The spectrum obtained for PAH-RB shows bands that can be assigned to amide bond between PAH and RB; *e.g.*, C–O stretching mode (amide I) at 1620 cm^−1^, and N–H in-plane bending mode (amide II)^39^ at 1535 cm^−1^. Amide I and amide II bands present in PAH-RB appeared with the concomitant disappearance of carboxylic acid peak at 1608 cm^−1^, indicating the amide bond formation with carboxylate group of RB. Additionally, the sharp peak corresponding to C–halogen stretching at 950 cm^−1^ is preserved but broadened after functionalization.

**Fig. 3:**
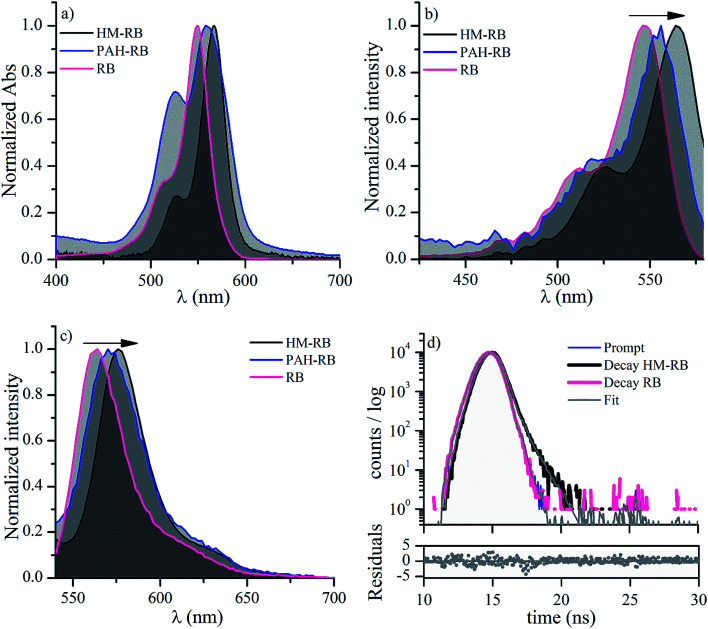
(a) UV-vis spectra (b) excitation fluorescence spectra (c) emission fluorescence spectra for RB in aqueous solution, PAH-RB and HM-RB in NaCl (50 mM) aqueous solution. (d) Emission fluorescence decays registered at 580 nm for HM-RB and free RB in air-equilibrated aqueous solutions.

The UV-vis absorption spectrum of PAH-RB in aqueous solutions was registered and compared with free RB (PAH is completely transparent in the visible region). PAH-RB spectrum displays one band in the green spectral region with well-defined maximum (*λ*_1_ = 558 nm) and a shoulder (*λ*_2_ = 523 nm), as expected for xanthene dyes (Fig. 3a).^40^ However, these bands appear broader and red-shifted in comparison to those of free RB. Such behavior can be associated with the self-aggregation of xanthene dyes resulting in a red shift of the absorption maxima and a decrease of the maximum-to-shoulder absorbance ratio (*A*_1_/*A*_2_).^40^ Hence, a comparison of normalized absorbance spectra of RB and PAH-RB in Fig. 3a suggests that aqueous solution of PAH-RB features the expected effect corresponding to dye–dye interactions in the polymeric structure.

The excitation/emission spectra of PAH-RB appear red-shifted compared to RB (Fig. 3b and c). Such spectral shifts are expected for RB in a less protic and polar environment.^41^ In addition, the fluorescence quantum yield (*Φ*_F_) value of PAH-RB is more than one-order of magnitude lower than that corresponding to free RB (Table 1), which can be explained assuming a strong static quenching of RB fluorescence as a consequence of the intense dye–dye interactions in the functionalized polymer. In time-resolved experiments, fluorescence lifetime (*τ*_F_) of RB in aqueous environments showed a mono-exponential decay with a *τ*_F_ 98 ± 10 ps, as expected for monomeric RB in water (see Fig. 3d).^42^ Although there is some minor proportion of non-aggregated PAH-RB responsible for the observed steady-state emission, such contribution is not enough to be determined in the time-resolved experiments.

**Table 1:** Summary of the results obtained for RB in aqueous medium, bound to the polymer (PAH-RB) and in hollow microcapsules (HM-RB)

Sample	*λ* ^abs^_max_ (nm) ± 2	*A*_1_/*A*_2_^a^ ± 0.2	*λ* ^emi^_max_ (nm) ± 2	*λ* ^exc^_max_ (nm) ± 2	*Φ*_F_	*τ*_F_ (ps) ± 10	*τ*_T_ (μs) ± 0.2	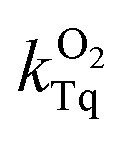 (M^−1^ s^−1^) × 10^9^ ± 0.02
RB	549	3.1	546	564	0.02^b^	98	60.0	1.50
PAH-RB	558	1.5	556	570	≤10^−3^	ND	14.0	0.40
HM-RB	568	3.7	564	577	≥0.4	27	0.9	0.97
						786	63.2	0.04

a Ratio of the absorbance at the maximum of the RB peak to the absorbance at the absorption shoulder.

b Quantum yield corresponds to RB in the monomeric form in solution.^41^

DLS analysis showed an increased hydrodynamic diameter from 122 ± 19 nm to 250 ± 50 nm, when comparing PAH and PAH-RB respectively. Considering that covalent binding of RB to PAH involve a negligible increase in average molecular weight, the observed size increment can be understood in terms of association of neighboring polymer chains caused by dye functionalization.

Zeta potential was determined for PAH and PAH-RB in aqueous solution (pH 7.0, [NaCl] = 500 mM) and the values obtained were +14.0 mV and +15.0 mV, respectively. The positive value of PAH-RB is crucial for the construction of LbL architectures. The fact that the same zeta potential value was determined for both systems is consistent with the low percentage of amine groups that reacts with RB. That is, the positive charge of PAH is due to the protonated amino groups and, according to the ratio of reactants used in the synthesis of PAH-RB (see Experimental section), the resulting fraction of amino groups linked to RB molecules is very low, which means that the decrease of the total charge of the polymer is negligible.

The above discussed results confirm that synthesized PAH-RB features covalently attached RB, but also suggest the association between dye molecules on neighboring polymeric chains, which are likely to be favored by hydrophobic interactions; and cause a strong effect on the spectroscopic properties of RB.

### Hollow PAH-RB microcapsules

**Fig. 4:**
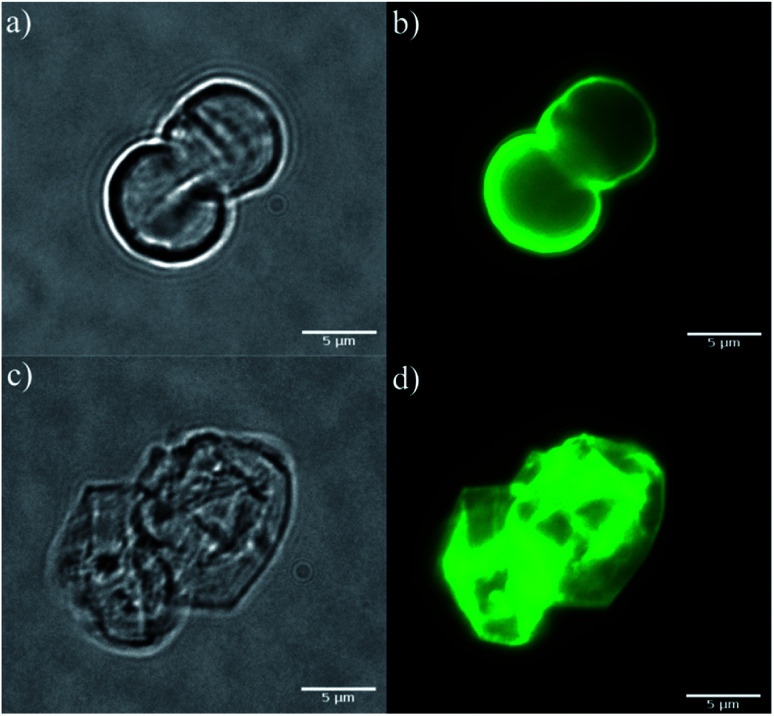
Optical (a and c) and fluorescence (b and d) microscopy images of microcapsules with CaCO_3_ (a and b) and after the dissolution of the mold with EDTA (c and d). Excitation was at 545 nm and emission was collected at 605 nm. The image color does not correspond to the light emitted by the sample. It was digitally colored post-acquisitions.

To evaluate the integrity of microcapsules constructed *via* LbL with PAH-RB and PSS, optical and fluorescence microscopy images were recorded before and after removal of CaCO_3_ template with EDTA. This procedure leads to the formation of hollow microcapsules, hereafter referred as HM-RB. As it can be observed in Fig. 4a and c, template removal did not significantly change the size of microcapsules, but affected the shape registered, which was due to the loss of the rigid filler material and was expected considering previous reports. In addition, microcapsules with and without CaCO_3_ core exhibit the characteristic fluorescence of RB dye upon excitation at 545 nm (Fig. 4b and d, respectively), thus confirming the presence of dye in the multilayered coating.

Fig. 3a also shows the UV-vis spectrum of HM-RB after baseline correction by scattering. RB spectrum when supported on HM features a red-shifted narrower band with *A*_1_/*A*_2_ ratio higher than the corresponding to PAH-RB (see Table 1). These spectral features strongly suggest that supramolecular organization after LbL formation hinders dye–dye interactions. Accordingly, both excitation and emission spectra of HM-RB suspensions showed red-shifted bands when compared to those corresponding to PAH-RB aqueous solutions (Fig. 3a and b). More importantly, *Φ*_F_ of HM-RB was determined to be twenty times greater than the corresponding to dye aqueous solution (Table 1). In time-resolved experiments, the fluorescence decay can be fitted assuming a bi-exponential decay with an increased *τ*_F_ value of 786 ± 10 ps (compared to 98 ps, Table 1), together with a 27% decrease on the additional decay process, as can be observed in Fig. 3d. This complex decay for HM-RB indicates that the behavior of dye singlet excited states is strongly influenced by the heterogeneous environment of LbL assembly, which is in line with the observed red shift in steady state fluorescence experiments (see Fig. 3c).

Spectral analysis of HM-RB suggests that LbL assembly on the CaCO_3_ template surface favors two effects. On the one hand the disaggregation of the dye, that is, the separation of the RB molecules, avoiding the interactions observed in PAH-RB. On the other hand the immobilization of the dye, similar to what already reported for proteins^43^ and other thin films.^44,45^ It is expected that both effects hinder the deactivation pathways of the excited states of the dye, which would enhance its photosensitizing properties. This point will be analyzed in detail in the next paragraphs.

It is known that triplet excited states play a dominant role in the initiation of photosensitized processes, therefore the formation of triplet RB (^3^RB*) species was investigated in both PAH-RB and HM-RB Ar-saturated aqueous suspensions by laser-flash photolysis experiments. In PAH-RB suspensions a mono-exponential decay of 14 μs was observed, almost 23% shorter than values corresponding to free RB. Differently, for HM-RB, the measured transient absorption changed into a bi-exponential decay featuring longer lifetime compared to PAH-RB and similar to free RB (within experimental error, see Table 1). The above described transients can be assigned to the triplet excited state of RB in different environments; based on the observed decay rates increase in presence of O_2_. The corresponding total bimolecular quenching rate constant 
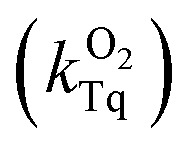
 was calculated according to the Stern–Volmer eqn (3), and the corresponding values are reported in Table 1 (Fig. S2†), where 
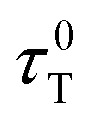
 and *τ*_T_ are the triplet lifetime of RB in the absence and in the presence of O_2_, respectively.3
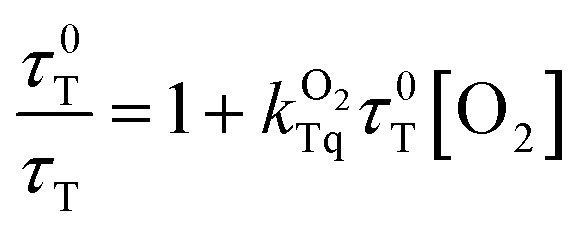


In this case, an increase of complexity in the assembly (from PAH-RB to HM-RB) leads to a decrease in 
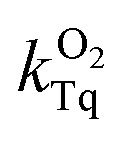
 value; which can be rationalized considering that O_2_ free diffusion is partially hindered in the heterogeneous assembled multilayer environment.

The results shown in this section suggest that RB molecules are accommodated into the microcapsule shell in a spatial arrangement that hinders dye–dye interaction. This supramolecular array is compatible with the observation that, upon irradiation, singlet and triplet excited states of RB (^1^RB* and ^3^RB*) are efficiently generated. The larger the local molecular rigidity around RB in the multilayer, the more favored an efficient radiative deactivation of ^1^RB*; thus, conferring an intense fluorescence in HM-RB suspensions. Furthermore, the formation of ^3^RB* suggests that HM-RB can feature photosensitizing properties, as it is analyzed in the next section.

### Photosensitizing properties of HM-RB

**Fig. 5:**
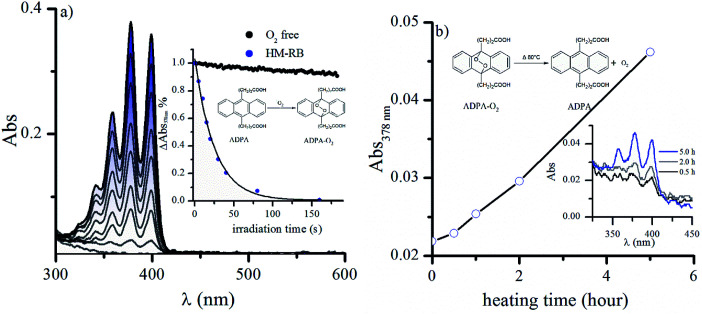
(a) Time-evolution of UV-vis spectra of ADPA in the presence of HM-RB under irradiation. Inset: normalized absorbance changes of ADPA at 378 nm *vs.* irradiation time for an air-equilibrated suspension of HM-RB (red), in comparison to the same sample in the absence of oxygen (black). (b) Time evolution of absorbance at 378 nm over different periods on anthracene endoperoxide thermolysis. Inset: UV-vis spectra of ADPA after different heating time at 80 °C. Irradiation was carried out with the irradiation setup I.

Although RB molecules remain immobilized into the multilayer of the HM-RB and generates ^3^RB* upon irradiation, which is quenched by molecular oxygen, it is not straightforward to assume that this supramolecular system can act as an efficient photosensitizer. Generation of ^1^O_2_ upon irradiation of HM-RB was detected using anthracene-9,10-dipropionic acid disodium salt (ADPA) acting as chemical-trap reaction of ^1^O_2_, since it becomes photo-bleached to give an endoperoxide.^46^Fig. 5a shows the UV-absorbance decreases of ADPA dissolved in the supernatant of HM-RB suspension as a function of the irradiation time using the irradiation setup I (see Experimental section). The first-order kinetic decay of ADPA relative absorbance at 378 nm is shown in Fig. 5a inset, confirming a constant steady-state concentration of ^1^O_2_. A similar significant decrease of ADPA absorbance was observed in HM-RB suspensions, as well as in aqueous RB solutions after 180 s irradiation (no ADPA consumption was observed in control experiments without irradiation).

In contrast, Ar-bubbling in HM-RB suspensions strongly reduces the consumption of ADPA upon illumination, suggesting that ADPA oxidation occurs by ^1^O_2_-mediation to yield the corresponding endoperoxide. To confirm such assumption, oxidized ADPA solutions were heated while the absorbance was monitored in order to provoke thermolysis of endoperoxides, thus regenerating the parent aromatic anthracene ring.^47^Fig. 5b shows the recovery of typical anthracene ring absorption bands upon heating at 80 °C. These results confirm the effective generation of ^1^O_2_ by irradiation with visible light of HM-RB suspensions.

### HM-RB as photosensitizer for the oxidation of dGMP

**Fig. 6:**
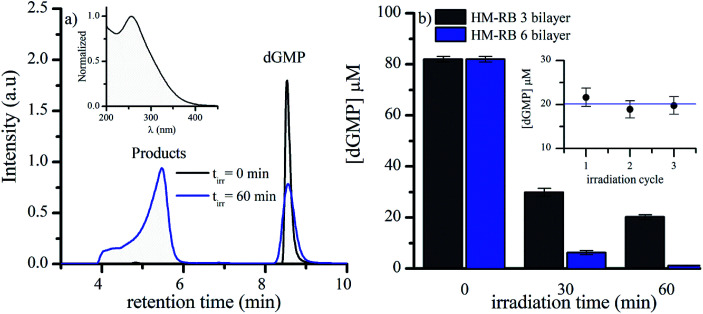
(a) Chromatograms of supernatant of air-equilibrated aqueous suspensions containing dGMP and HM-RB, recorded using the UV/vis detector, before and after 60 min of irradiation. Inset: normalized absorption spectra of oxidation product of retention time 5.5 min. [dGMP]_0_ = 80 μM, pH = 6.5, *λ*_irr_ = 567 nm, *λ*_an_ = 260 nm. (b) Concentration profiles of dGMP in aqueous suspension of HM-RB with different number of bilayers at different irradiation time. Inset: dGMP concentration after 60 min of consecutive irradiation cycles. pH = 7, *λ*_irr_ = 567 nm, irradiation set up II.

The possible application of polymeric photosensitizers bearing RB chromophores in microcapsules was tested with the model reaction of 2′-deoxyguanosine 5′-monophosphate (dGMP) degradation as a biological target. We chose this nucleotide because in DNA guanine is the only nucleobase that reacts significantly with ^1^O_2_.^48^ Therefore, aqueous suspensions of HM-RB and dGMP were exposed to UV-vis radiation using the irradiation setup II (see Experimental section). Supernatants obtained from the irradiated samples were analyzed by HPLC using the UV/vis detector. The decrease observed for peak area corresponding to dGMP was used as an indicator of nucleotide consumption in the process. In addition, several peaks with shorter retention times (*t*_r_) were detected (Fig. 6a). It was found that HM-RB featuring 3 or 6 bilayers of polyelectrolytes can effectively photosensitize dGMP degradation (Fig. 6b). This result suggests that O_2_ can diffuse across the HM-shells with different number of bilayers and interact with ^3^RB* to generate ^1^O_2_, that ultimately will produce the observed substrate oxidation.

Taking into account previous results, 2′-deoxyspiroimidantoin 5′-monophosphate (dSpMP) was identified as the primary product of ^1^O_2_ mediated reaction.^49^ Analyzing the UV spectra and *t*_r_ of the reaction products using irradiated HM-RB and dGMP suspension, the principal product identity can be assigned to dSpMP, which suggests the participation of ^1^O_2_ in the reaction mechanism. Additionally, an increase of dGMP consumption was observed as pH was increased from 7 to 9 (Fig. S3†). It is noteworthy that dGMP chemical reactivity towards ^1^O_2_ is higher as pH becomes alkaline, which confirms that photosensitization mechanism of dGMP by HM-RB is ^1^O_2_ mediated.

The dGMP consumption after 60 min of irradiation in successive irradiation-centrifugation cycles for HM-RB and dGMP suspensions was evaluated. For each subsequent irradiation experiment, pellets were re-suspended in dGMP fresh solutions. It is possible to reuse the same HM-RB batch since dGMP consumption was proportional to the irradiation time, and additionally, similar consumption was obtained for at least three irradiation cycles of 60 minutes each (Fig. 6b inset). This behavior showed two interesting advantages of HM-RB over RB in solution. First, the supramolecular organization prevents the dye photobleaching reactions, and second HM-RB can be easily separated by centrifugation from the reaction mixture to be reused. On the contrary, RB solution requires more sophisticated and expensive separation techniques such as chromatography.

## Conclusions

In the present work we have presented a strategy for the assembly of hollow microcapsules (HM) using sacrificial CaCO_3_ templates, *via* LbL approach constituted by PSS (sodium poly(styrene sulfonate)) polyanion and a modified polycation, PAH-RB, (poly(allylamine hydrochloride)) covalently modified with rose Bengal dye. The presence of attached bulky aromatic RB molecule on modified PAH induces dye–dye interactions, presumably driven by hydrophobicity. Such aggregation modifies supported RB both, spectroscopic and photochemical properties, reducing its fluorescence quantum yield (*Φ*_F_). The spectroscopic analysis of HM-RB revealed that the observed behavior corresponds to what can be expected for free RB in less protic and polar environments, without significant dye–dye interactions.

**Scheme 2:**
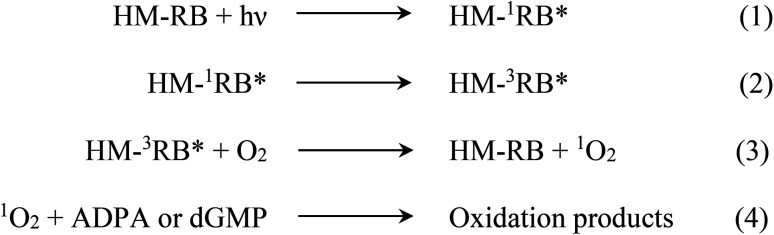
Degradation mechanism of ADPA or dGMP photosensitized by HM-RB.

Irradiation with visible light of HM-RB aqueous suspensions induces the efficient production of singlet oxygen (^1^O_2_) comparable to what produced by RB in aqueous solutions. Such reactive oxygen species was demonstrated to be responsible for degradation of a biological target molecule used (2′-deoxyguanosine 5′-monophosphate, dGMP). A proposed degradation mechanism is described in Scheme 2 (reactions (1)–(4)). After subsequent irradiation cycles and oxidation of dGMP, the reutilization of HM-RB as photosensitizer was also proved indicating that the supramolecular organization of the RB molecules into the HM shell prevents the photobleaching reactions induced by dye self-interactions.

Summarizing, we have demonstrated that by controlling the supramolecular assembly and spatial arrangement of RB attached to a polymeric structure; the photophysical and photochemical characteristics of the dye can be modulated, remarkably recovering photosensitization properties of RB in homogeneous solution with the advantage of high photostability and an easier separation for its subsequent use.

## Conflicts of interest

There are no conflicts to declare.

## Supplementary Material

RA-009-C9RA03153G-s001
