# Supplementary material for: Photosensitizing properties of hollow microcapsules built by multilayer self-assembly of poly(allylamine hydrochloride) modified with rose Bengal
Source: RSC Adv. 2019 Jun 18;9(33):19226–35. doi: 10.1039/c9ra03153g (PMC9065151; doi:10.1039/c9ra03153g)
Supplement: RA-009-C9RA03153G-s001 [file RA-009-C9RA03153G-s001.pdf]

Electronic Supplementary Material (ESI) for RSC Advances.
This journal is © The Royal Society of Chemistry 2019

Electronic Supplementary Information (ESI)

Photosensitizing properties of hollow microcapsules built by multilayer self-assembly of poly(allylamine hydrochloride) modified with rose Bengal

Mariana P. Serrano[1], Matías Rafti[1], Andrés H. Thomas[1] and Claudio D. Borsarelli[2]*

1 Instituto de Investigaciones Fisicoquímicas Teóricas y Aplicadas (INIFTA), Departamento de Química, Facultad de Ciencias Exactas, Universidad Nacional de La Plata, CCT La Plata-CONICET, La Plata, Argentina

2 Instituto de Bionanotecnología del NOA (INBIONATEC), CONICET. Universidad Nacional de Santiago del Estero (UNSE), RN9, Km 1125, G4206XCP Santiago del Estero, Argentina.

* Corresponding author email: mariserr@inifta.unlp.edu.ar

Synthesis of PAH-RB polycation

3 mM of 1-ethyl-3-(3-dimethylamino-propyl) carbodiimide (EDC), solution was added to a Rose Bengal (RB) buffered 2-(4-morpholino)-ethane sulfonic acid (MES) (10 mM, pH 6.0) solution (30 µM, 10 mL) and stirred evenly. Then, 0.4 mM of N-hydroxysulfo succinimide sodium salt (sulfo-NHS) solution was added and stirred for 30 minutes in order to achieve carboxyl activation. Finally, 20 mL of poly(allylamine hydrochloride) (PAH) (7.5 mg/mL) in buffer HEPES solution (50 mM, pH 7.2) was added and stirred for 15 h. The product was purified by dialysis during 48 h with 12 KDa MW (molecular weight cut-off). The whole reaction and dialysis process were protected from light.

Hollow microcapsule preparation

1 mL of 0.25 M $CaCl_2$ ultra-pure deionized water solution was placed in 2 mL Eppendorfs and kept at 25 °C under 800 rpm orbital shaking. Afterwards, 500 µL of 0.5 M Na_2CO_3 aqueous solution were added and stirred for 30 s. Immediately, the suspension was centrifuged at 1500×*g* during 60 s and three-times washed with water, followed by a final wash with pure ethanol. The centrifuged pellet was dried at 35 °C overnight, and the powder was stored in dry ambient until further use.

Polymeric microcapsules were built using $CaCO_3$ templates sequentially coated with oppositely charged polyelectrolyte layers (LbL assembly) Briefly; i) 30 mg of $CaCO_3$ microparticles were suspended with 1 mL of PEI (1 mg/mL) solution and placed in an ultrasonic bath for 60 s until complete dispersion; ii) the suspension was placed in the mixer during 15 min at 25 °C with shaking at 1100 rpm; and finally iii) the suspension was centrifuged at 1500×*g* for 60 s, the supernatant removed and the pellets re-suspended with 1 mL of 50 mM NaCl solution.

The washing cycle was repeated twice. Afterwards, PEI coated microparticles were suspended with 1 mL of PSS solution (1 mg/mL) and steps i) to iii) were repeated. The next layer was placed by adsorbing PAH-RB (1mg/ml) using the same protocol. The multilayers were formed by alternating adsorption until reaching the desired number of bilayers of anionic and cationic polyelectrolytes for the microparticles. UV-vis spectra were recorded for the supernatant after each polyelectrolyte addition (PSS or PAH-RB) in order to follow the formation of subsequent layers. For the described procedure, approximately 70% of the PAH-RB remains surface-adsorbed, and such adsorption efficiency was maintained throughout the successive layer depositions (Figure S1).

Removal of $CaCO_3$ template was carried by re-suspension of the coated microparticles with 0.1 M EDTA solution at pH 7 during 30 min at 25 °C while shaking at 1100 rpm. The ions resulting from $CaCO_3$ dissolved template were separated by centrifugation at 6000×g for 15 minutes and then the supernatant removed. The described EDTA treatment was repeated two-times to ensure complete removal of $CaCO_3$. A final wash was performed using 50 mM NaCl solution to remove the remaining EDTA (Scheme 1). Finally, the obtained HM were stored at 8°C before use.

Additional experiments on HM assembly, quenching of the triplet excited state of PAH-RB, and dGMP consumption as detected via HPLC.

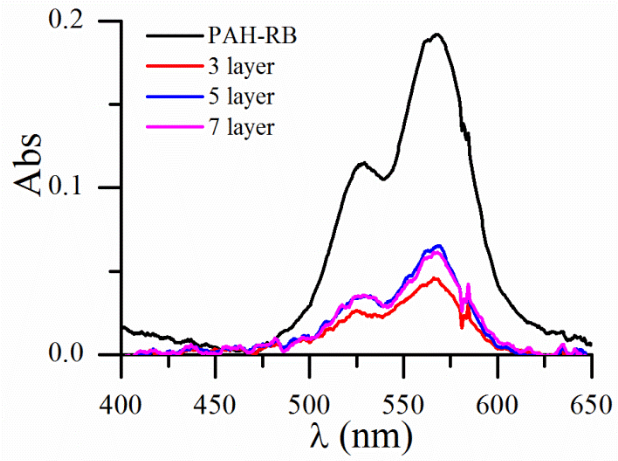

Figure S1. UV-vis spectra of PAH-RB registered for the supernatant after each successive addition of PAH-RB layer during multilayer self-assembly process.

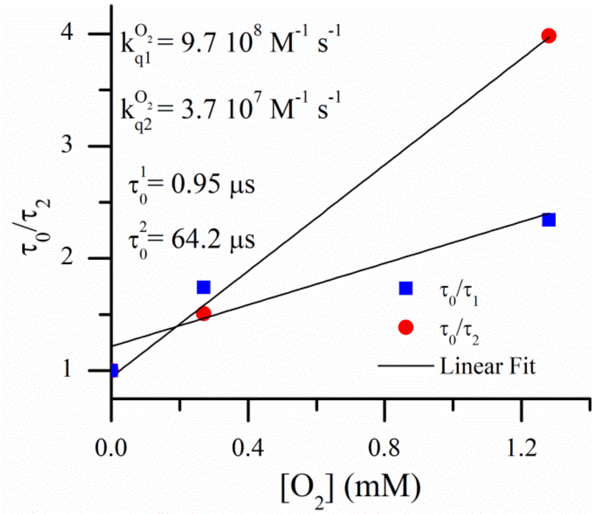

Figure S2. Stern–Volmer plot of the quenching of the triplet excited state of PAH-RB in hollow microcapsules by dissolved O_2. The triplet lifetime values were calculated analyzing the transient decay at 560 nm.

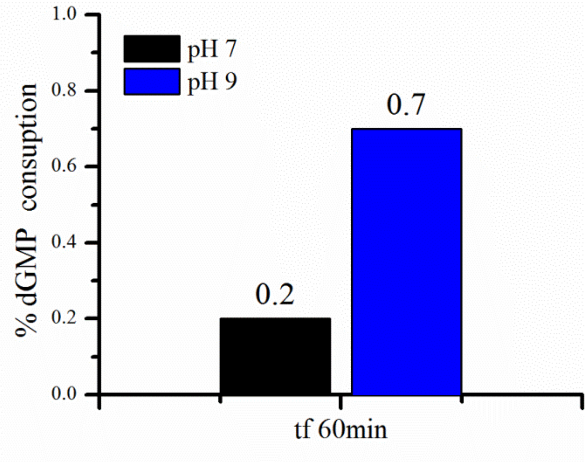

Figure S3. Comparison of dGMP consumption in aqueous suspension of hollow microcapsules after 60 minutes of irradiation time in neutral an alkaline media. $[dGMP]_0$ =80 μM, λ_{irr}=567 nm.
